# Risk Factors for Hospital Readmission after Radical Gastrectomy for Gastric Cancer: A Prospective Study

**DOI:** 10.1371/journal.pone.0125572

**Published:** 2015-04-27

**Authors:** Cheng-Le Zhuang, Su-Lin Wang, Dong-Dong Huang, Wen-Yang Pang, Neng Lou, Bi-Cheng Chen, Xiao-Lei Chen, Zhen Yu, Xian Shen

**Affiliations:** 1 Department of Gastrointestinal Surgery, The First Affiliated Hospital, Wenzhou Medical University, Wenzhou, China; 2 Wenzhou Key Laboratory of Surgery, The First Affiliated Hospital, Wenzhou Medical University, Wenzhou, China; 3 Department of Gastrointestinal Surgery, Shanghai Tenth People’s Hospital Affiliated to Tongji University, Shanghai, China; University of Florida, UNITED STATES

## Abstract

**Background:**

Hospital readmission is gathering increasing attention as a measure of health care quality and a potential cost-saving target. The purpose of this prospective study was to determine risk factors for readmission within 30 days of discharge after gastrectomy for patients with gastric cancer.

**Methods:**

We conducted a prospective study of patients undergoing radical gastrectomy for gastric cancer from October 2013 to November 2014 in our institution. The incidence, cause and risk factors for 30-day readmission were determined.

**Results:**

A total of 376 patients were included in our analysis without loss in follow-up. The 30-day readmission rate after radical gastrectomy for gastric cancer was 7.2% (27of 376). The most common cause for readmission included gastrointestinal complications and postoperative infections. On the basis of multivariate logistic regression analysis, preoperative nutritional risk screening 2002 score ≥ 3 was an independent risk factor for 30-day readmission. Factors not associated with a higher readmission rate included a history of a major postoperative complication during the index hospitalization, prolonged primary length of hospital stay after surgery, a history of previous abdominal surgery, advanced age, body mass index, pre-existing cardiopulmonary comorbidities, American Society of Anesthesiology grade, type of resection, extent of node dissection and discharge disposition.

**Conclusions:**

Readmission within 30 days of discharge after radical gastrectomy for gastric cancer is common. Patients with nutritional risk preoperatively are at high risk for 30-day readmission. Preoperative optimization of nutritional status of patients at nutritional risk may effectively decrease readmission rates.

## Introduction

Readmission of patients to the hospital after major surgery is a common event. Nearly one in seven patients hospitalized for a major surgical procedure is readmitted to the hospital within 30 days after discharge [1]. In the United States, the cost to Medicare of unplanned readmissions in 2004 was 17.4 billion dollars [2]. Owing to its enormous medical and financial impacts, readmission is gathering increasing attention as a measure of health care quality as well as a potential cost-saving target [3].

Gastric cancer is the fourth most common cancer and the second leading cause of cancer death globally, with almost 1 million new cases estimated to occur annually [4]. The highest incidence rates are in Eastern Asia, Eastern Europe, and South America [4]. Surgical resection remains the most effective therapy for potentially curable gastric cancer [5]. However, gastrectomy is associated with high complications rates and long hospitalizations [6, 7].

Several studies have attempted to identify risk factors for readmission after abdominal surgery in general [8], colorectal [9–12] and hepato-pancreato-biliary surgery [13–15], however, there is a paucity of information that focuses on readmission rates and associated risk factors for readmission after gastrectomy for patients with gastric cancer. Gastrectomy is frequently performed in malnourished patients, which can be associated with a higher incidence of postoperative complications [16]. A retrospective study identified that a history of a major postoperative complication was independently associated with a higher 30-day readmission rate in patients underwent curative gastrectomy for gastric cancer [17]. The purpose of this prospective study was to ascertain the incidence of readmission and examine whether preoperative nutritional risk is an independent risk factor for readmissions within 30 days of discharge after gastrectomy for gastric cancer.

## Patients and Methods

In this prospective study, patients undergoing elective gastrectomy for gastric cancer at the Department of Gastrointestinal Surgery, The First Affiliated Hospital of Wenzhou Medical University from October 2013 to November 2014 were included. Patients undergoing gastrectomy with palliative intent or patients with age < 18 years were excluded from the study. In addition, patients who died during their primary hospitalization after surgery or died within 30 days of discharge were also excluded from this study. Admissions were screened independently by 2 surgeons (C.L.Z. and S.L.W.). Discrepancies were solved by consensus, and there was an adjudicator (X.S) in case of persistent disagreement. Readmission was defined as admission to the inpatient service within 30 days of discharge from hospital. We chose the period of 30 days because the 30-day readmission rate has long been the standard rate reported in previous studies. When more than 1 reason for readmission was found for 1 patient, only the most significant problem was considered as the reason for readmission. All participants provided their written informed consent and the protocol for this prospective study was approved by the ethics committee of The First Affiliated Hospital of Wenzhou Medical University.

Patients were followed up every 10 days by phone calls until 30 days after discharge. The follow-up was conducted by a surgeon (W.Y.P). In addition, patients were arranged to visit the operating surgeon in the out-patient within 2 weeks after discharge. For each patient, the following parameters were collected: 1) patient characteristics, including age, gender, TNM stage, body mass index (BMI), American Society of Anesthesiology (ASA) grade, pre-existing cardiopulmonary comorbidities, diabetes, preoperative nutritional risk score, history of previous abdominal surgery; 2) operative details, including operative method, type of resection, extent of node dissection, type of reconstruction, surgical duration; 3) postoperative outcomes, including postoperative complications during the index hospitalization, length of postoperative hospital stay, readmissions within 30 days of discharge; 4) discharge disposition, including those who were discharged to home with home services, discharged to home without services, discharged to rehabilitation centers; 5) the reasons for readmission and the types of treatment. Preoperative nutritional risk assessment was performed within 24 h of admission using the nutritional risk screening (NRS) 2002, patients with a total score of 3 or more were considered at nutritional risk [18]. Major complications were defined as those meeting the criterion of grade II or higher according to the Clavien-Dindo classification [19].

To identify risk factors independently associated with readmission within 30 days of discharge, variables including patient characteristics, operative details, postoperative outcomes and discharge disposition were first subjected to univariate analysis (chisquare tests for categorical variables and Student’s independent t-test for continuous variables), determining the relationship of each variable to 30-day readmission. This was followed by a multivariate logistic regression model, which included all of the variables with a p value of < 0.10. All statistical analyses were performed using SPSS version 22.0 (IBM SPSS Inc).

## Results

Initially, 378 patients undergoing gastrectomy for gastric cancer met the inclusion criteria. Two patients died during the index hospitalization after surgery. Finally, a total of 376 patients were included in the analysis without loss in follow-up. Patient characteristics are listed in Table 1. The median age of patients was 65.5 years, and most of patients (76.9%) were male. The median length of postoperative hospital stay was 13 days. For discharge disposition, 67 patients were discharged to home and serviced by family, 309 patients were discharged to home without services, and no patient was discharged to rehabilitation centers. Overall, 27 patients were readmitted within 30 days of discharge for a readmission rate of 7.2%. All these 27 patients were readmitted in our hospital. The median time to readmission was 11 days after discharge. The median length of hospital stay for readmission was 13 days. No patient was admitted more than once within 30 days of discharge after gastrectomy.

**Table 1:** Characteristics of the Patients.

Characteristic	No. of patients^a^
Age (years)^b^	65.5 (31–87)
Sex	
Male	289 (76.9)
Female	87 (23.1)
BMI (kg/m^2^)^b^	22.06 (15.06–32.11)
NRS2002 score	
< 3	254 (67.6)
≥ 3	122 (32.4)
ASA grade	
I	19 (5.1)
II	304 (80.9)
III	53 (14.1)
Operation method	
Laparoscopy	40 (10.6)
Open	336 (89.4)
Type of resection	
Total gastrectomy	137 (36.4)
Subtotal gastrectomy	239 (63.6)
Extent of node dissection	
D0	20 (5.3)
D1	71 (18.9)
D2	284 (75.5)
D3	1 (0.3)
Type of reconstruction	
Billroth I	142 (37.8)
Billroth II	90 (23.9)
Roux-en-Y	144 (38.3)
TNM stage	
I	139 (37.0)
II	55 (14.6)
III	174 (46.3)
IV	8 (2.1)

^a^ Values in parentheses are percentages unless indicated otherwise

^b^ Values are median (range)

The causes of readmission of the 27 patients were diverse and are summarized in Table 2. The most common cause for readmission was delayed gastric emptying, accounting for 5 of 27 (18.5%) readmissions. On readmission, 23 of these patients were managed conservatively; however, 3 patients with anastomotic leakage, gastrointestinal hemorrhage and intra-abdominal hemorrhage, respectively, underwent surgical interventions. One patient with anastomotic stricture underwent endoscopic balloon dilation. Two of these 27 patients died during their rehospitalization. One patient died of bowel obstruction and the other one died of sepsis.

**Table 2:** Causes of Readmission in 27 Patients after Gastrectomy for Gastric Cancer.

Causes of Readmission	No. of patients
Delayed gastric emptying	5
Intra-abdominal fluid collection	3
Cerebral infarction	3
Wound infection	2
Fever	2
Pneumonia	2
Diarrhea	2
Gastrointestinal hemorrhage	2
Intra-abdominal hemorrhage	1
Anastomotic leakage	1
Anastomotic stricture	1
Small bowel obstruction	1
Abdominal pain	1
Dumping syndrome	1
Total	27

Univariate analysis of variables associated with readmission is presented in Table 3. Patients who had a history of a major postoperative complication during the index hospitalization were significantly more likely to be readmitted (*P* = 0.039). In addition, NRS 2002 score ≥ 3 was significantly associated with an increased risk of readmission (*P* = 0.008). However, advanced age, BMI, pre-existing cardiopulmonary comorbidities, ASA grade ≥ III, type of resection, extent of node dissection did not predict an increased risk of readmission. Other possible factors like gender, diabetes, a history of previous abdominal surgery, primary length of postoperative hospital stay, and discharge disposition were also not significant factors for readmission. However, on multivariate logistic regression analysis (Table 4), a history of a major postoperative complication during the index hospitalization did not remain a significant impact on the rate of readmission. Finally, NRS 2002 score ≥ 3 was an independent risk factor for 30-day readmission based on multivariate analysis (*P* = 0.01). In further analysis, NRS 2002 score ≥ 3 was associated with a higher incidence of major postoperative complications (P = 0.038). The individual data points behind means, medians and variance measures presented in the results and tables are available in S1 Dataset.

**Table 3:** Univariate analysis of factors associated with 30-day readmission.

Factors	Readmission (n = 27)	No readmission (n = 349)	Odds ratio	95% CI	*P* value
Age					
< 75	21	286	1		
≥ 75	6	63	1.297	0.503–3.345	0.590
Sex					
Female	7	80	1		
Male	20	269	0.850	0.347–2.082	0.721
BMI					0.254
< 18.5	5	37			
18.5–25	20	254			
> 25	2	58			
NRS 2002 score					
< 3	12	242	1		
≥ 3	15	107	2.827	1.280–6.245	0.008*
ASA grade					0.318
I	3	16			
II	20	284			
III	4	49			
Operation method					
Open	22	314	1		
Laparoscopy	5	35	2.039	0.727–5.722	0.168
Type of resection					
Subtotal gastrectomy	20	219	1		
Total gastrectomy	7	130	0.590	0.243–1.432	0.239
Extent of node dissection					0.971
D0	1	19			
D1	5	66			
D2	21	263			
D3	0	1			
Type of reconstruction					0.488
Roux-en-Y	8	136			
Billroth I	13	129			
Billroth II	6	84			
TNM stage					0.607
I	12	127			
II	2	53			
III	12	162			
IV	1	7			
Cardiopulmonary comorbidity					
No	19	235	1		
Yes	8	114	0.868	0.369–2.043	0.746
Diabetes					
No	25	310	1		
Yes	2	39	0.636	0.145–2.789	0.545
Previous abdominal surgery					
No	24	317	1		
Yes	3	32	1.238	0.353–4.340	0.738
Surgical duration					
< 3.5 hours	13	228	1		
≥ 3.5 hours	14	121	2.029	0.924–4.455	0.073
Length of postoperative stay ≥ 13 days					
No	17	166	1		
Yes	10	183	0.534	0.238–1.198	0.123
Major postoperative complication					
No	19	298	1		
Yes	8	51	2.460	1.021–5.919	0.039*
Discharge disposition					
Discharged to home and serviced by family	6	61	1		
Discharged to home without services	21	288	0.741	0.287–1.914	0.535

* Statistically significant

**Table 4:** Multivariate logistic regression analysis of factors associated with 30-day readmission.

Factors	Odds ratio	95% CI	*P* value
NRS 2002 score ≥ 3	2.827	1.280–6.245	0.010

## Discussion

In 2009, an article in the New England Journal of Medicine reported that 19.6% of all hospitalized Medicare patients were readmitted within 30 days of discharge [2]. One year after that, the Affordable Care Act of 2010 was passed and the Hospital Readmissions Reduction Program was established in the United States [8]. Since then, strategies to reduce the rate of readmissions are gathering increasing attention. However, from 2006 to 2011, all-cause readmission rates have only decreased from 16.0% to 15.3% [17]. Recently, several studies focusing on readmission rates in patients undergoing colorectal [9–12] and hepato-pancreato-biliary surgery [13–15] have been reported. Gastrectomy is complex and associated with high rates of postoperative morbidity and mortality, however, few studies examining the rates of readmission and associated risk factors for readmission in patients undergoing gastrectomy have been published. In the present study, the 30-day readmission rate after radical gastrectomy for gastric cancer was 7.2%. The median length of hospital stay for readmission was 13 days, which was same to the primary length of stay after surgery. On multivariate logistic regression analysis, we found that preoperative NRS 2002 score ≥ 3 was an independent risk factor for 30-day readmission. This finding is important and meaningful because NRS 2002 score ≥ 3 is a preventable risk factor before surgery.

There have been 2 retrospective studies reporting the rates of readmission and associated risk factors for readmission in patients undergoing gastrectomy [17, 20]. Ahmad and colleagues reported a 14.6% 30-day readmission rate after curative gastrectomy for gastric cancer, and identified that type of resection, pre-existing cardiovascular disease and a history of a major postoperative complication were independently associated with a higher 30-day readmission rate [17]. However, the patients included in this study spanned a time period of more than 15 years, and most patients with advanced gastric cancer underwent D0 or D1 gastrectomy, but not D2 gastrectomy [17]. Another study was conducted by Kim and colleagues in Korea, and they reported a 7.5% 30-day readmission rate after radical subtotal gastrectomy for patients with early gastric cancer. The longer primary hospital stay after surgery was the only risk factor for readmission in their study [20]. However, compared with Korea, patients with early gastric cancer account for a small proportion in China and West countries, and the conclusions from patients with early gastric cancer cannot be generalized to patients with advanced gastric cancer.

To our knowledge, the present study is the first prospective study reporting the rates of readmission and associated risk factors for readmission after curative gastrectomy for gastric cancer. In the present study, patients with early or advanced gastric cancer were included, and the treatment for gastric cancer was based on the Japanese Gastric Cancer Treatment Guidelines (ver. 3) [21]. In addition, the period of this study was only 13 months. Therefore, the results from the present study were strengthened and can be generalized to early and advanced gastric cancer.

Malnutrition is very common in patients with gastric cancer. It was reported that the prevalence of nutritional risk in patients with gastric cancer ranges from 36% to 43% [22, 23], and malnutrition was associated with a higher incidence of postoperative complications in patients underwent major abdominal surgery [24, 25]. However, for gastrectomy, the relationship between postoperative complications and readmission remains controversial in previous studies [17, 20]. In addition, there were no reports regarding the relationship between nutritional risk and readmission rates in patients undergoing gastrectomy. In the present study, NRS 2002 score was first introduced as a potential risk factor for readmission. We found that 122 of 376 patients (32.4%) were at nutritional risk, which was consistent with the reports from other studies [22, 23]. Univariate analysis showed that a history of a major postoperative complication and NRS 2002 score ≥ 3 were risk factors associated with a higher 30-day readmission rate. However, in the multivariate analysis, a history of a major postoperative complication did not remain significant, and NRS 2002 score ≥ 3 was the only risk factor for 30-day readmission in patients undergoing gastrectomy. In addition, our results also showed that NRS 2002 score ≥ 3 was associated with a higher incidence of major postoperative complications, which confirmed the findings of previous studies. Perhaps this explains the fact that a history of a major postoperative complication is not an independent risk factor for 30-day readmission based on multivariate analysis.

The most common reason for readmission in our study is gastrointestinal complications (delayed gastric emptying, gastrointestinal hemorrhage, diarrhea, small bowel obstruction, anastomotic leakage, anastomotic stricture and dumping syndrome), followed by postoperative infectious complications (wound infection, pneumonia, fever, etc.). This finding is consistent with the 2 previous studies [17, 20]. Patients with nutritional risk preoperatively are susceptible to gastrointestinal complications, because patients with nutritional risk have a lower tolerance for malnutrition caused by gastrointestinal complications. In addition, it was reported that malnutrition was associated a higher incidence of postoperative infectious complications [24, 25]. Taken together, these may explain the main finding of the present study that preoperative NRS 2002 score ≥ 3 was an independent risk factor for 30-day readmission in patients undergoing gastrectomy. Therefore, it is critical that surgeons optimize the nutritional status of patients at nutritional risk before surgery. A minimum of 7 days of parenteral nutrition before surgery is recommended for patients with severe malnutrition who cannot be adequately orally or enterally fed [26]. For patients who have a good tolerance of enteral nutrition, oral or enteral (nasogastric or nasojejunal tube) feeding is recommended [16]. During the postoperative period, parenteral nutrition or enteral nutrition is required for patients who are unable to achieve 60% of their requirements [16]. These approaches may play an important role in the prevention of readmission.

It was controversial whether a prolonged primary length of hospital stay after operation was a risk factor for readmission in the 2 previous studies [17, 20]. Kim and colleagues found that the longer primary hospital stay after surgery was a statistically significant predictor of readmission [20]. However, Ahmad and colleagues reported that this factor did not have a significant impact on the rate of readmission [17]. In the present study, we examined the primary length of stay after surgery as both a categorical variable (using 13 days as a cut-off) and a continuous variable, and in neither case did longer length of stay prove to be a risk factor for 30-day readmission.

It remains controversial in the treatment of gastric cancer between West and East [27]. In the West, although D2 lymphadenectomy is recommended, most surgeons are still adopting D1 lymphadenectomy [17, 28, 29]. In the East, however, surgeons routinely perform D2 lymphadenectomy for patients with gastric cancer [21]. As for chemotherapy, preoperative neoadjuvant chemotherapy is widely used in the West [17, 27–29], while in the East, preoperative chemotherapy is rarely used [21]. In the present study, only 2 patients received preoperative chemotherapy. Due to these different treatment approaches between West and East, our results may not be generalized to the West. Besides, it is noteworthy that preoperative chemotherapy is associated with a higher incidence of malnutrition. As preoperative chemotherapy is a widely accepted treatment approach for patients with advanced gastric cancer in the West, it is worthwhile to investigate the relationship between preoperative nutritional risk and readmission in western countries.

Our study has several limitations. First, it is a single institution study, although the sample size was not small. A multicenter prospective study is essential to overcome this limitation. Second, our data only support conclusions regarding risk factors for 30-day readmission. Long-term follow-up data are needed in future study to investigate the relationship between readmission rates and mortality in patients undergoing gastrectomy. Third, as enhanced recovery after surgery (ERAS) programs are not implemented in our department, so the findings may not be generalizable to patients undergoing gastrectomy in the context of ERAS programs.

In conclusion, based on this large prospective study, the 30-day readmission rate after radical gastrectomy for gastric cancer is 7.2%. We demonstrate that preoperative NRS 2002 score ≥ 3 is an independent risk factor for 30-day readmission. Gastrointestinal complications and postoperative infections are the common causes for readmission. Preoperative optimization of nutritional status of patients at nutritional risk may effectively decrease readmission rates.

## Supporting Information

S1 Dataset The individual data points behind means, medians and variance measures presented in the results and tables. (XLSX)
